# Advanced diffusion imaging in grey matter reflects individual differences in cognitive ability in older adults

**DOI:** 10.1162/IMAG.a.1035

**Published:** 2025-11-20

**Authors:** Adam Kimbler, Craig E.L. Stark

**Affiliations:** Department of Neurobiology and Behavior, University of California, Irvine, CA, United States

**Keywords:** diffusion, NODDI, mean apparent propagator MRI, tensors

## Abstract

Diffusion-weighted imaging is a tool that can non-invasively provide insights into the microstructure of a given brain region. Various advanced techniques exist within the diffusion-weighted imaging space that each provides valuable insights into different aspects of microstructure. In the following study, we sought to examine whether the combination of derived diffusion metrics (tensors, neurite orientation dispersion and density imaging (NODDI), and mean apparent propagator (MAP) MRI) in grey-matter regions could reliably predict cognitive performance in older adults, and whether these findings were replicable across datasets. First, we demonstrated that all combinations of diffusion metrics could reliably determine participant characteristics and were significant predictors of age. Second, we found that a combination of Tensor, NODDI, and MAP-MRI metrics within the hippocampus could predict Rey Auditory Verbal Learning Task (RAVLT) performance in older adults above and beyond any combination of two metrics alone. We also found these diffusion metrics were able to reliably predict RAVLT performance and Trails B performance, but not performance on a One-Back working memory task. We also found that these same combinations of metrics could predict working memory performance, but not memory performance within a region associated with working memory (right hemisphere nucleus accumbens). Taken together, these findings indicate that these diffusion metrics provide valuable information on grey-matter microstructure independent of one another, and that the ability to obtain both NODDI and MAP-MRI-based information from multi-shell diffusion scans more than justifies additional scan time.

## 1 Introduction

Magnetic resonance imaging (MRI) is an invaluable tool for detecting differences in the brain that underly behavioral, cognition, and pathology. Diffusion-weighted imaging, a subcategory of MRI that focuses on the diffusion of water molecules across the brain (Stejskal & Tanner, 1965), has frequently been used as a tool to examine the underlying microstructure of the brain, often highlighting the role of white-matter tracts in brain function. Diffusion tensor metrics and tractography make the assessment of axonal integrity connectivity between regions relatively straightforward (Assaf & Pasternak, 2008; Madden et al., 2012; Sasson et al., 2010; Thomason & Thompson, 2011). The detection of such minute changes in structure is crucial for the understanding and treatment of different types of pathology, such as Alzheimer’s (Weston et al., 2015) and Parkinson’s diseases (Y. Zhang & Burock, 2020). While most studies tend to focus on changes in white-matter structure using diffusion MRI, research into grey-matter microstructure to predict cognition has made great strides in recent years (Aggarwal et al., 2015; Assaf, 2019; Budde & Annese, 2013; Colgan et al., 2016; Radhakrishnan et al., 2020, 2022; Venkatesh et al., 2020)

More advanced techniques such as neurite orientation and dispersion and density imaging (NODDI: H. Zhang et al., 2012) can help elucidate the different microstructural components of cells within a given voxel, providing us the estimates of the extracellular and intracellular components and the free water diffusing within the voxel. A more recent tool within diffusion MRI is Mean Apparent Propagator (MAP) MRI (Özarslan et al., 2013), which represents 3D q-space via an analytical series expansion, allowing for use to understand both Gaussian and non-Gaussian diffusion in complex tissue microstructures, allowing previously unanalyzed microstructures to be mapped. While previous work from our laboratory and others has shown that NODDI metrics in grey matter can be used to predict cognitive performance above and beyond tensors and volume alone (Kamiya et al., 2020; Radhakrishnan et al., 2022), and other work has demonstrated that regional MAP-MRI metrics can predict memory performance on their own (Singh et al., 2024), no study has examined whether combinations of these tensor, NODDI, and MAP-MRI metrics provide an increase in prediction of memory performance.

In the current study, we sought to examine whether diffusion-derived metrics (tensors, NODDI, MAPMRI) in grey matter can be used to predict cognitive performance in older adults. We sought to replicate previous findings from our laboratory indicating that hippocampal diffusion metrics can predict age in both our previously used dataset and an additional dataset (ADNI). Further, we sought to replicate our findings that hippocampal diffusion can predict memory performance on a memory task across multiple datasets, and whether the addition of MAP-MRI-based metrics can provide additional predictive utility above and beyond tensors and NODDI alone. Finally, we sought to extend this by assessing whether there is a structure–function relationship present such that hippocampal diffusion metrics would be specifically related to memory tasks and whether other regions’ diffusion metrics might predict individual differences in other cognitive domains.

## 2 Methods

### 2.1 Participants

Participants in UC, Riverside (UCR) dataset consisted of 71 older adults recruited from a study at the University of California, Riverside (Radhakrishnan et al., 2022). We then filtered out subject IDs that had a Rey Auditory Verbal Learning delay score of less than 5 (n = 15). The final sample consisted of 56 older adults (73.51 ± 6.31 years, 42 female). All participants provided informed consent before participation in this study and were compensated for their time. All experimental procedures were approved by the University of California, Riverside Review Board.

Participants in the ADNI consisted of 223 older adults from the Alzheimer’s Disease Neuroimaging Initiative 3 (ADNI3) dataset that had an available multi-shell diffusion MRI acquisition obtained on a Siemens scanner. The data were accessed and downloaded on January 22^nd^, 2024. After obtaining this initial dataset, we filtered out subjects who did not have either the RAVLT or Cogstate Brief Battery information available (n = 26). We then filtered out all subject IDs that had a diagnosis other than cognitively normal (n = 86) to keep the data in line with that acquired in the UCR dataset. Finally, to further keep the datasets similar, we then filtered out subject IDs that had a Rey Auditory Verbal Learning delay score of less than 5 (n = 32). Additionally, one subject was excluded due to low-quality segmentation. This resulted in a final sample of 78 subjects (age = 73.13 ± 8.22 years, 52 female).

### 2.2 MR acquisition

#### 2.2.1 UCR

Data were acquired on Siemens Prisma 3T MRI scanner (Siemens Healthineers, Malvern, PA) with a 32-channel receive-only head coil. Head movements were minimized with fitted padding.

##### 2.2.1.1 T1w

Structural images were acquired using a magnetization-prepared rapid gradient echo (MP-RAGE) protocol (echo time (TE)/repetition time (TR) = 2.72/2400 ms, 208 axial slices, FOV = 300 x 320 mm, GRAPPA acceleration factor = 2, and in 0.8 mm isotropic resolution) that resulted in a T1-weighted image.

##### 2.2.1.2 DWI

Two multi-shell (6 0 s/mm^2^, 64 1500 s/mm^2^, 64 3000 s/mm^2^ shells) sequences were acquired with the following diffusion parameters: TE/TR = 102/3500 ms, FOV = 212 × 182 mm, 64 axial slices, acquisition time = 10 min and 57 s, multi-band acceleration factor = 4 and in 1.7 mm isotropic resolution. Each acquisition was acquired on the axial plane, with the first acquisition being in the anterior-posterior direction and the second in the posterior-anterior direction.

#### 2.2.2 ADNI

##### 2.2.2.1 T1w

Structural images were acquired using a magnetization-prepared rapid gradient echo (MP-RAGE) protocol (echo time (TE)/repetition time (TR) = 2.98/2300 ms, 208 axial slices, FOV = 240 x 256 mm, GRAPPA acceleration factor = 2, and in 1.0 mm isotropic resolution) that resulted in a T1-weighted image.

##### 2.2.2.2 DWI

One multi-shell (13 0 s/mm^2^, 6 500 s/mm^2^, 48 1000 s/mm^2^, and 60 2000 s/mm^2^ shells) sequence was available with the following diffusion parameters: TE/TR = 71/3400 ms, FOV = 116 × 116 mm, 81 axial slices, acquisition time = 10 min and 57 s, multi-band acceleration factor = 3 and in 2.0 mm isotropic resolution. The acquisition was acquired on the axial plane with a phase-encoding direction of posterior-anterior.

### 2.3 Anatomical data preprocessing

The T1-weighted (T1w) image was corrected for intensity non-uniformity (INU) using N4BiasFieldCorrection (Tustison et al., 2010, ANTs 2.3.1) and used as T1w-reference throughout the workflow. The T1w-reference was then skull stripped using antsBrainExtraction.sh (ANTs 2.3.1), using OASIS as target template. Spatial normalization to the ICBM 152 Nonlinear Asymmetrical template version 2009c (Fonov et al., 2009, RRID:SCR_008796) was performed through nonlinear registration with antsRegistration (ANTs 2.3.1, RRID:SCR_004757, Avants et al., 2008), using brain-extracted versions of both T1w volume and template. Brain tissue segmentation of cerebrospinal fluid (CSF), white matter (WM), and grey matter (GM) was performed on the brain-extracted T1w using FAST (FSL 6.0.3:b862cdd5, RRID:SCR_002823, Y. Zhang et al., 2001).

### 2.4 Diffusion data preprocessing

Images were grouped into two phases encoding polarity groups. Any images with a b-value less than 100 s/mm^2^ were treated as a *b* = 0 image. MP-PCA denoising as implemented in MRtrix3’s dwidenoise (Veraart et al., 2016) was applied with a 5-voxel window. After MP-PCA, Gibbs unringing was performed using MRtrix3’s mrdegibbs (Kellner et al., 2016). Following unringing, B1 field inhomogeneity was corrected using dwibiascorrect from MRtrix3 with the N4 algorithm (Tustison et al., 2010). After B1 bias correction, the mean intensity of the DWI series was adjusted so all the mean intensity of the b = 0 images matched across each separate DWI scanning sequence. Both distortion groups were then merged into a single file, as required for the FSL workflows. After B1 bias correction, the mean intensity of the DWI series was adjusted so all the mean intensity of the b = 0 images matched across each separate DWI scanning sequence. Both distortion groups were then merged into a single file, as required for the FSL workflows.

FSL (version 6.0.3:b862cdd5)’s eddy was used for head motion correction and Eddy current correction (Andersson & Sotiropoulos, 2016). Eddy was configured with a *q*-space smoothing factor of 10, a total of 5 iterations, and 1000 voxels used to estimate hyperparameters. A linear first level model and a linear second level model were used to characterize Eddy current-related spatial distortion. *q*-Space coordinates were forcefully assigned to shells. Field offset was attempted to be separated from subject movement. Shells were aligned post-eddy. Eddy’s outlier replacement was run (Andersson et al., 2016). Data were grouped by slice, only including values from slices determined to contain at least 250 intracerebral voxels. Groups deviating more than 4 standard deviations from the prediction had their data replaced with imputed values. Data were collected with reversed phase-encode blips, resulting in pairs of images with distortions going in opposite directions. Here, multiple DWI series were acquired with opposite phase encoding directions, so b = 0 images were extracted from each. From these pairs, the susceptibility-induced off-resonance field was estimated using a method like that described in Andersson et al. (2003). The fieldmaps were ultimately incorporated into the Eddy current and head motion correction interpolation. Final interpolation was performed using the Jacobian modulation method to account for signal pile-up/dilution caused by local stretching/compression (Andersson & Sotiropoulos, 2015).

Several confounding time series were calculated based on the preprocessed DWI: framewise displacement (FD) using the implementation in *Nipype* (following the definitions by Power et al. (2014). The head motion estimates calculated in the correction step were also placed within the corresponding confounds file. Slice-wise cross-correlation was also calculated. The DWI time series were resampled to ACPC, generating a *preprocessed DWI run in ACPC space* with 2 mm isotropic voxels.

Many internal operations of *QSIPrep* use *Nilearn* 0.8.1 (Abraham et al., 2014, RRID:SCR_001362) and *Dipy* (Garyfallidis et al., 2014). For more details of the pipeline, see the section corresponding to workflows in *QSIPrep*’s documentation.

### 2.5 Diffusion reconstruction

Reconstruction was performed using *QSIprep* 0.14.3, which is based on *Nipype* 1.6.1 (Gorgolewski et al., 2011, 2018; RRID:SCR_002502).

#### 2.5.1 DSI studio reconstruction

Diffusion orientation distribution functions (ODFs) were reconstructed using generalized q-sampling imaging (GQI, Yeh et al., 2010) with a ratio of mean diffusion distance of 1.25. Three tensor values were computed from this fitting that were used in subsequent analyses: axial diffusivity (AD), generalized fractional anisotropy (gFA), and radial diffusivity (RD).

#### 2.5.2 NODDI reconstruction

The NODDI model (H. Zhang et al., 2012) was fitted using the AMICO implementation (Daducci et al., 2015). A parallel diffusivity value of 1.3 µm^2^/ms and an isotropic diffusivity value of 3.0 µm^2^/ms were used. The parallel diffusivity value was chosen based on previous work finding differences in parallel diffusivity between grey and white matter (Guerrero et al., 2019). This model fit was then used to compute three outcome metrics: orientation dispersion index (ODI), neurite density index (NDI), and free-water fraction (FWF).

#### 2.5.3 MAP-MRI reconstruction

The MAP-MRI model (Özarslan et al., 2013) was fitted using the DIPY (Garyfallidis et al., 2014) implementation using default values. Default values of DIPY were used, with the following parameters being set: Radial Order = 6, Laplacian Regularization = True, Laplacian Weighting = 0.2, Positivity Constraint = True, Global Constraints = False, pos grid = 15, pos radius = adaptive, Anisotropic Scaling = True, Eigenvalue Threshold = 0.0001, bval threshold = infinite, dti scale estimation = True, static diffusivity = 0.0007, cvxpy solver = None). Three MAPMRI metrics were used due to their relative independence of correlation mean square diffusivity (MSD), q-space inverse variance (QIV), return-to-origin probability (RTOP), and non-Gaussianity (NG).

### 2.6 Region-of-interest (ROI) masking

ROI masks were calculated by transforming the Brainnetome (Fan et al., 2016) 246 region atlas from MNI space to subject-specific anatomical space through the usage of antsApplyTransforms (Avants et al., 2008) and extracting data for each metric (volume, AD, gFA, RD, ODI, NDI, FWF, MSD, QIV, RTOP, NG) for each ROI. Hippocampal ROI analyses included all four hippocampal ROIs from the atlas (Caudal and Rostral Hippocampus for both hemispheres).

### 2.7 Cognitive testing

Participants included in both the UCR and ADNI datasets were administered the Rey Auditory Verbal Learning Test (RAVLT) (Rey, 1941). This measure consists of three parts: An initial five presentations of the same 15-word list with the participant being asked to immediately recall as many items as possible from the list, a second immediate recall test after being administered an interference list of 15 different words, and a delayed recall test administered 15 min after the second immediate recall test. RAVLT Delay score represents performance on the final part, which is a scale from 0 to 15 based on the number of recalled words. For later analysis, a binarized version of this delay score is used, with individuals with a score greater than 9 assigned as 1, and those less than or equal to a 9 receiving a 0.

Participants included in ADNI had several additional metrics available, notably the Trail Making Test and the Cogstate Brief Battery. The Trail Making Test Consists of two parts: Part A requires participants to connect circles with numbers in numerical order, while Part B has them connecting alternating numbers and letters in ascending order. The results of the participants’ performance on Part B were used as an outcome metric in the current study. The Cogstate Brief Battery (CBB) consists of a 15-min assessment that assesses attention, visual learning, and working memory. It provides four tests: Detection, Identification, One Card Learning, and One-Back performance. We opted to use the three tests that were not directly associated with semantic memory performance: Detection, Identification, and One-Back.

### 2.8 Logistic regression analysis

For each combination of predictors, a logistic regression analysis was conducted in Scikit-Learn (1.3.0, Pedregosa et al., 2011) with fivefold cross-validation using an L2 penalty function (Ridge regression) to reduce the risk of overfitting. For analyses focused on the hippocampus, estimates for each metric from each of the four hippocampal ROIs were used, resulting in 4 x the number of metrics for each analysis being predictors in the model (e.g., volume alone results in 4 predictors, DTI+NODDI+MAP results in 4 x 9 predictors for a total of 36 predictors in the model). For models not predicting binarized age, age was included as an additional controlled variable in each model.

## 3 Results

### 3.1 Hippocampal diffusion metrics easily predict age in older adults

As an initial test of the viability of grey-matter diffusion for classification, we median split each dataset by age, replicating the method used in Radhakrishnan et al. (2022). All participants were over age 53 years, but this splits them into relatively older and younger groups (UCR: 68.38 vs. 78.15 mean age; ADNI: 67.30 vs. 80.94 mean age). We then performed a logistic regression analysis using hippocampal diffusion metrics to classify age groups.

**Fig. 1.**
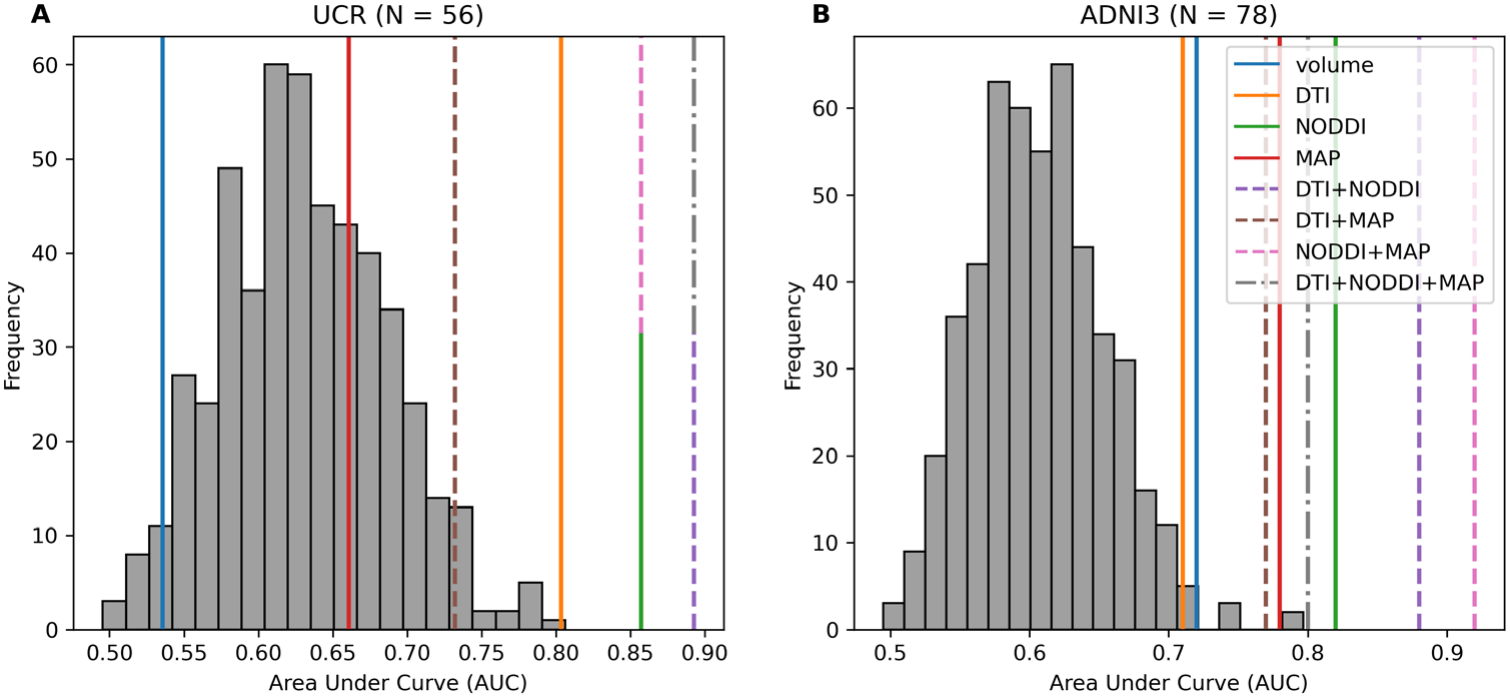
(A) Within the UCR dataset, 5 metrics significantly predicted binarized age via median split with a maximum AUC of .892 for DTI+NODDI+MAP and DTI+NODDI and a minimum AUC of .536 for volume. (B) Within the ADNI dataset, all metrics significantly predicted binarized age, with a maximum AUC of .924 for NODDI+MAP and a minimum AUC of .708 for DTI. Metric sets alone are represented by solid lines, combinations of two metric sets are represented by a dashed line, and combinations of three metric sets are represented by a dotted and dashed line. Metric sets with the same AUC are represented by a stacked line.

Within the UCR dataset, six sets of metrics were significant predictors of binarized median-split age in older adults: DTI+NODDI (AUC = .892, p < .001), DTI+NODDI+MAP (AUC = .892, p < .001), NODDI (AUC = .857, p < .001), NODDI+MAP (AUC = .857, p < .001), DTI (AUC = .804, p = .002), and DTI+MAP (AUC = .732, p = .022) (Fig. 1A). Similar results were present in ADNI, with all eight sets of metrics being significant predictors of binarized age: NODDI+MAP (AUC = .924, p < .001), DTI+NODDI (AUC = .884, p < .001), NODDI (AUC = .819, p < .001), DTI+NODDI+MAP (AUC = .805, p < .001), MAP (AUC = .776, p < .001), DTI+MAP (AUC = .765, p < .001), volume (AUC = .719, p = .004), and DTI (AUC = .708, p = .006) (Fig. 1B).

AUCs appeared to be relatively similar across datasets, with a few metric combinations failing to reach significance within the UCR dataset, likely due to the reduced sample size relative to ADNI. These results appear to be consistent with previous work that examined these combinations of metrics, which found that DTI+NODDI combinations provided increased predictive power compared with either metric alone. MAP does not appear to contribute much additional information across datasets, with DTI+NODDI+MAP showing equivalent results to DTI+NODDI within the UCR dataset and substantially lower AUC within ADNI (See Supplementary Tables 1 and 2 for an in-depth breakdown of model coefficients across datasets). For additional information on how diffusion metrics predict linear, rather than binarized age, see Supplementary Table 3.

### 3.2 A combination of hippocampal DTI, NODDI, and MAPMRI metrics strongly predicts RAVLT performance in older adults but differed across datasets

**Fig. 2.**
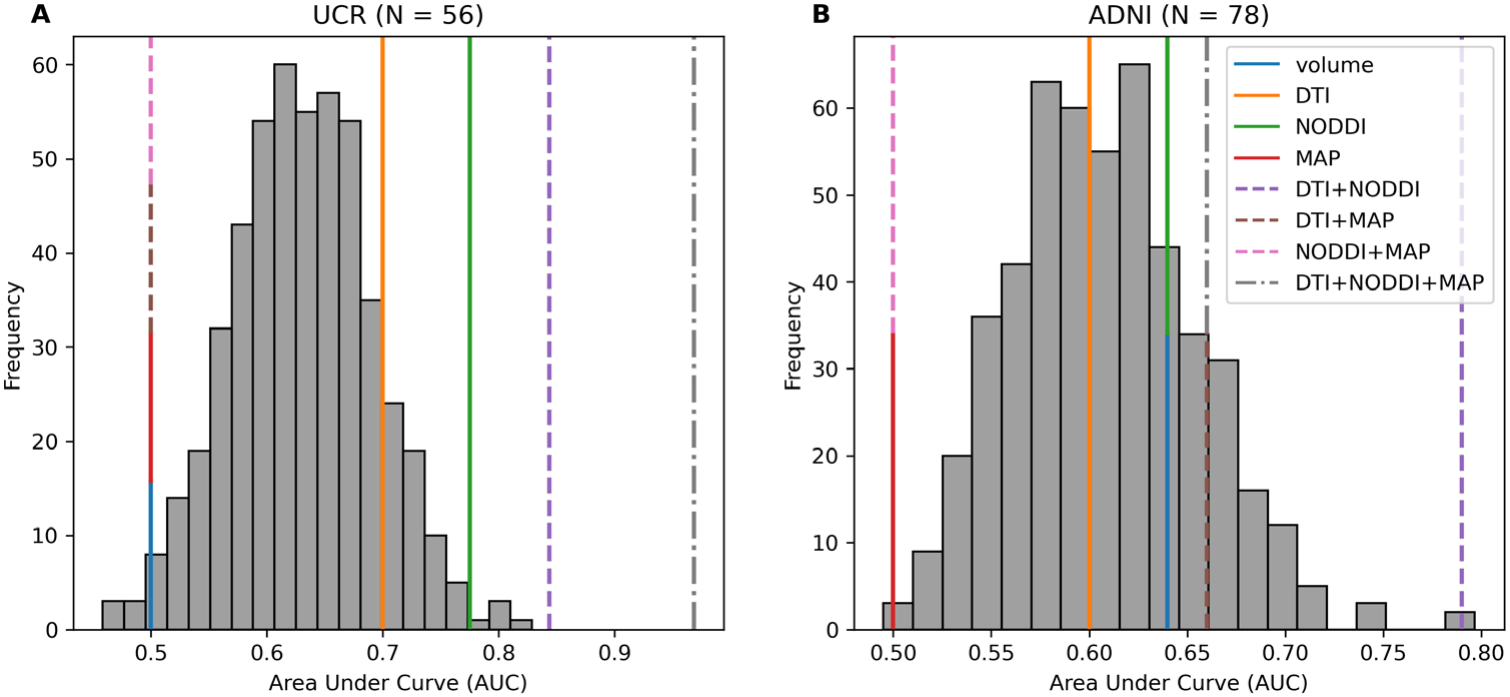
(A) Within the UCR dataset, several combinations of metrics were significant predictors of binarized RAVLT performance (DTI+NODDI+MAP: AUC = .969, p < .001; DTI+NODDI: AUC = .844, p < .001; NODDI: AUC = .775, p = .010). (B) Within ADNI, only one combination significantly predicted binarized RAVLT performance (DTI+NODDI: AUC = .790, p = .002). Metric sets alone are represented by solid lines, combinations of two metric sets are represented by a dashed line, and combinations of three metric sets are represented by a dotted and dashed line. Metric sets with the same AUC are represented by a stacked line.

We then conducted a similar analysis using hippocampal diffusion metrics to predict binarized RAVLT performance, while controlling for the age of the participants. Within the UCR dataset, three combinations of predictors significantly predict binarized RAVLT performance: DTI+NODDI+MAP (AUC = .969, p < .001), DTI+NODDI (AUC = .844, p < .001), and NODDI (AUC = .775, p = .010) (Fig. 2A). Fewer combinations significantly predict RAVLT performance in ADNI, with only one combination being significant: DTI+NODDI (AUC = .790, p = .002) (Fig. 2B). Across both datasets, DTI+NODDI was a strong set of predictors, with MAP substantially enhancing the predictive power of the model within the UCR dataset. This finding of the DTI+NODDI combination having a higher AUC than any other set of predictors alone is consistent with findings from our lab’s previous work (Radhakrishnan et al., 2022). MAP appears to have additional contributions beyond DTI+NODDI alone, but the power of the model appears to differ across datasets, potentially due to differences in acquisition (See Supplementary Tables 4 and 5 for an in-depth breakdown of model coefficients for each dataset.)

### 3.3 Hippocampal diffusion predicts performance on a range of explicit memory tasks

**Fig. 3.**
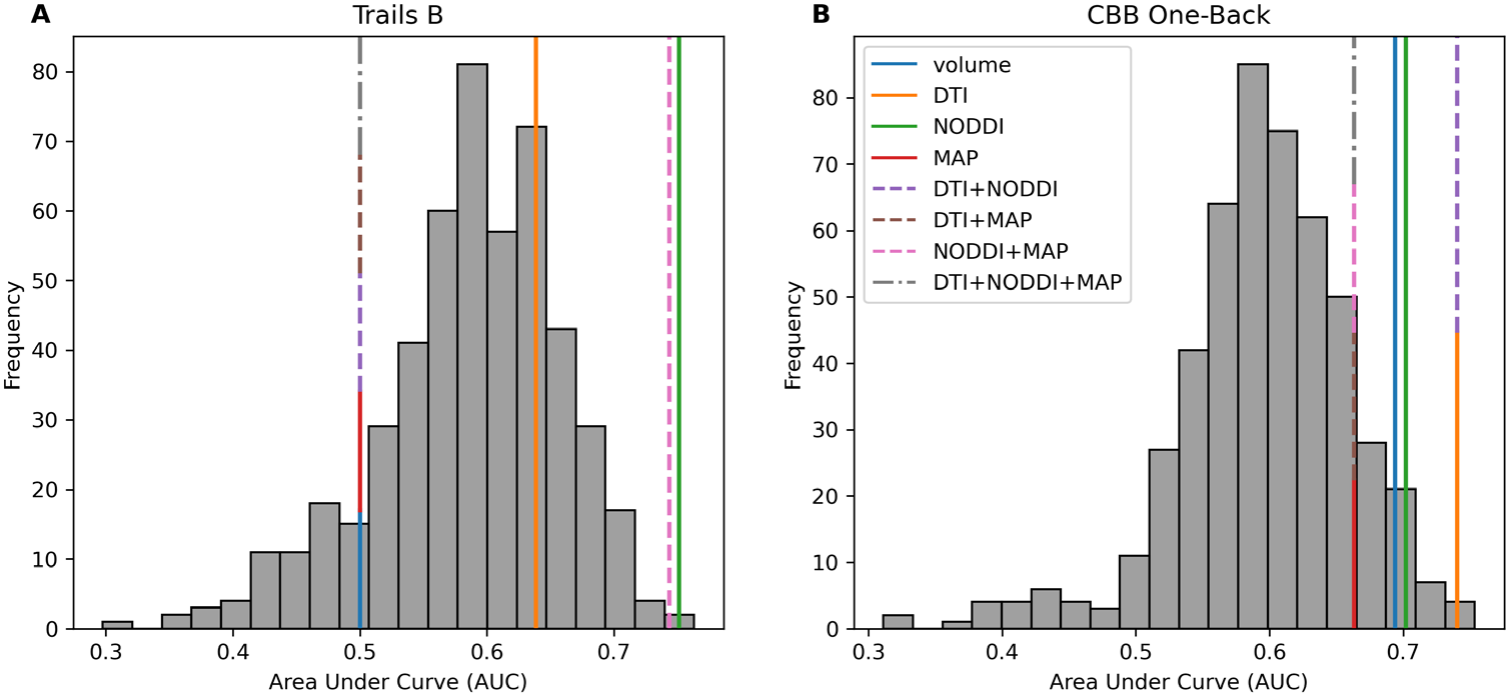
(A) Within the ADNI dataset, hippocampal NODDI and NODDI+MAP predicted Trails B performance (NODDI: AUC = .751, p = .002; NODDI+MAP: AUC = .744, p = .002). (B) Hippocampal diffusion metrics were also significant predictors of CBB One-Back performance (DTI: AUC = .740, p = .004; DTI+NODDI: AUC = .740, p = .004; NODDI: AUC = .702, p = .028; volume: AUC = .694, p = .048). Metric sets alone are represented by solid lines, combinations of two metric sets are represented by a dashed line, and combinations of three metric sets are represented by a dotted and dashed line. Metric sets with the same AUC are represented by a stacked line.

To examine whether hippocampal diffusion predicted memory performance specific to the RAVLT or just predicted memory performance in general, we followed similar procedures to analyze how hippocampal diffusion metrics predicted Trails B memory performance and performance on the CBB One-Back. For this analysis, we focused on ADNI, which contained performance metrics for RAVLT, Trails B, and CBB One-Back for all participants. We found that hippocampal NODDI (AUC = .751, p = .002) and NODDI+MAP (AUC = .744, p = .002) were significant predictors of Trails B performance (Fig. 3A). Hippocampal DTI (AUC = .740, p = .004), DTI+NODDI (AUC = .740, p = .004), NODDI (AUC = .702, p = .028), and volume (AUC = .694, p = .048) were also significant predictors of CBB One-Back (Fig. 3B). These findings indicate that these hippocampal diffusion metrics can predict memory performance across a myriad of tasks and are not limited in specificity to the RAVLT (See Supplementary Tables 6 and 7 for an in-depth breakdown of model coefficients).

### 3.4 Regional diffusion is sensitive to regional differences in task demands

**Fig. 4.**
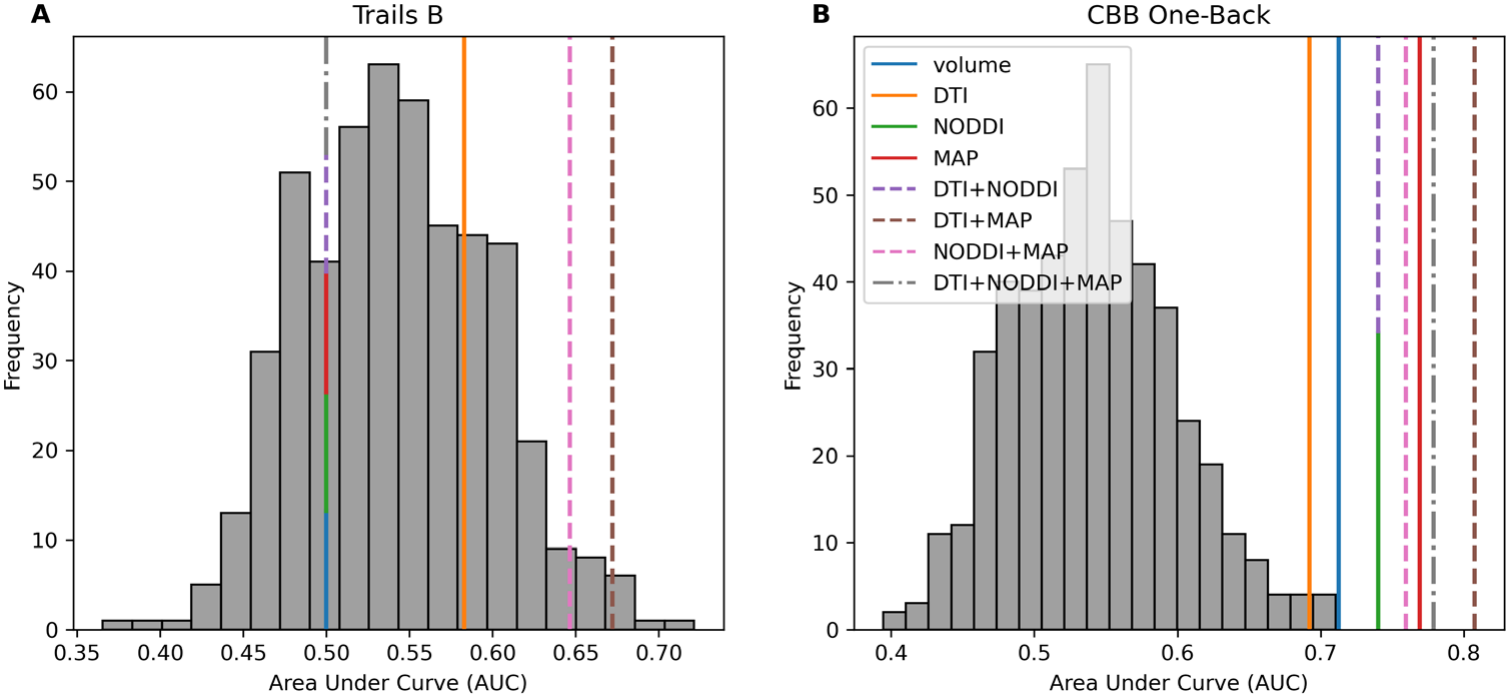
(A) Within the ADNI dataset, we identified an ROI (Right Nucleus Accumbens: NAC_R) with diffusion metrics that did not appear to predict memory performance on the RAVLT (with DTI+NODDI+MAP being the highest AUC at .664). (B) This ROI was able to strongly predict working memory performance on the One-Back (DTI+MAP: AUC = .808, p < .001; DTI+NODDI+MAP: AUC = .779, p < .001). Metric sets alone are represented by solid lines, combinations of two metric sets are Metric sets alone that are represented by solid lines, combinations of two metric sets are represented by a dashed line, and combinations of three metric sets are represented by a dotted and dashed line. Metric sets with the same AUC are represented by a stacked line.

To determine whether the ability of grey-matter diffusion to reflect cognition was a more general property, we conducted a search procedure across ROIs present in the Brainnetome atlas (Fan et al., 2016) to identify whether any of the ROIs present predicted working memory performance while not predicting memory performance on the RAVLT. One such ROI was identified (right hemisphere nucleus accumbens; represented as NAC_R in the Brainnetome Atlas). This region was not a significant predictor of memory performance on the RAVLT, with the strongest predictors being volume (AUC = .664, p > .05) (Fig. 4A). Diffusion from this region was, however, a strong predictor of CBB One-Back performance, with the strongest set of predictors being the combination of DTI+MAP (AUC = .808, p < .001) and DTI+NODDI+MAP (AUC = .779, p < .001) (Fig. 4B). This indicates that in addition to the sensitivity to specific task demands evidenced in the hippocampus, regional diffusion metrics are also sensitive to different aspects of cognition unique to each region, with at least one region being sensitive to working memory performance but not memory performance in general. Together, these findings represent a double dissociation (See Supplementary Tables 8 and 9 for an in-depth breakdown of model coefficients).

## 4 Discussion

In the current study, we demonstrated that grey-matter multi-shell diffusion metrics such as NODDI and MAP-MRI can provide significant benefits in predicting age-related changes in microstructure in older adults. We reproduced our previous results indicating that a combination of DTI+NODDI predicts age and explicit memory better than either metric alone, generalized these findings across two distinct datasets, and expanded this finding to include MAPMRI and found that combinations of three kinds of diffusion metrics (DTI+NODDI+MAP) were able to predict both age and memory performance above any combination of two metrics alone.

Consistent with previous work from our laboratory (Radhakrishnan et al., 2022), we found that all types of diffusion metric (Tensor, NODDI, MAPMRI) from within the hippocampus were strong predictors of age within both of our datasets and were all substantially better predictors than regional volume alone. The finding that even basic information on grey-matter microstructure provided by diffusion imaging being sensitive to age is consistent with previous work by other researchers as well (Aggarwal et al., 2015; Assaf, 2019; Leuze et al., 2014; Truong et al., 2014). Despite the relationships between the types of diffusion metrics, the increase in AUC as more metrics are added is evidence that each of these metrics is conveying at least somewhat distinct information about the hippocampus. Interestingly, despite using a different segmentation of the hippocampus relative to our previous work (our current rostral/caudal segmentation vs. specific subfields in Radhakrishnan et al., 2022), we found consistent results with predicting age and memory performance. This might suggest that the level of detail provided by hippocampal segmentation might be lost somewhat when working at lower resolutions with diffusion MRI.

We also were able to provide evidence of double dissociation within our work. We were able to show that hippocampal diffusion metrics were able to predict memory performance on the RAVLT and Trails B and that this prediction of cognitive performance did not generalize to other cognitive performance such as a one-back task. Further, we found that this pattern was differentiable across regions. Several regions were able to predict other cognitive task performance independent of each other, such as the nucleus accumbens being able to predict working memory performance but not performance on the RAVLT. This association with the nucleus accumbens and working memory performance is well known (see Silva et al., 2018 for a review). This finding provides even more evidence of the predictive utility of grey-matter diffusion for cognitive and diagnostic purposes.

Our current study has several important limitations. The first limitation is that our data across both samples represent a cross-section of the population. It is possible that while age is differentiable cross-sectionally within these datasets, that within individuals these changes in metrics over time might not be related in the same ways. As more data are acquired with multi-shell diffusion in datasets such as ADNI with multiple time points, this issue can be rectified with longitudinal analysis strategies. Another limitation, as outlined in our previous work, is that we binarized our outcome variables for age and memory performance. While this helps reduce noise and sample bias, we cannot conclusively say whether these same relationships would be observed when predicting absolute age or cognitive performance. Another limitation comes in the form of the complexity of the models (with up to 36 variables being included simultaneously in some models). These models show complex changes and divergences based on the presence of other regions and predictors in the model and even diverge based on laterality. Future studies would do well to use a form of variable selection or attempt dimensionality reduction beyond what is offered by Ridge regression to analyze how these models can be made simpler and reduce the burden of sampling size. A final limitation to note regards the need for multi-shell diffusion data for advanced diffusion analyses, and the difficulty in acquiring data across large datasets with similar imaging parameters. The need for multi-shell diffusion severely limited the availability of public datasets for use in our study, but with the optimization of multi-shell protocols to be included in future ADNI releases somewhat alleviates this problem. The differences in acquisition parameters, such as the overall shells and the magnitude of shells, can have a significant impact on the results within voxels, especially those with many crossing fibers, so DTI, NODDI, or MAP metrics could potentially vary based on differences in those parameters across datasets (Fritz et al., 2019).

In conclusion, multi-shell diffusion provides us with a treasure trove of important information on the microstructure of grey matter in the human brain. We have demonstrated that these metrics can be used separately or in tandem to predict both age and cognition in older adults, and that the microstructure of different regions predicts different types of cognitive performance. These metrics provide much value above and beyond volume or tensor-only diffusion and more than justify the extra time required to obtain multi-shell diffusion data.

## Supplementary Material

Supplementary Material

## Data Availability

Raw and preprocessed data supporting the findings will be made available upon request, made via e-mail to author A.K. or author C.S. The code used for analysis is available here: https://github.com/StarkLabUCI/greymatter_diffusion/
