# Supplementary material for: Advanced diffusion imaging in grey matter reflects individual differences in cognitive ability in older adults
Source: Imaging Neurosci (Camb). 2025 Nov 20;3:IMAG.a.1035. doi: 10.1162/IMAG.a.1035 (PMC12635482; doi:10.1162/IMAG.a.1035)
Supplement: Supplementary Material [file IMAG.a.1035_supp.pdf]

			DTI			NODDI			MAP			
Predictor	Auc	Region	AD	GFA	RD	ODI	NDI	FWF	MSD	QIV	RTOP	NG
DTI+NODDI	0.893	cHipp$_L$	-1.381	-0.699	-1.336	-1.096	-2.983	1.913				
		cHipp$_R$	0.683	-0.321	-0.898	-2.729	0.513	-2.649				
		rHipp$_L$	-1.892	-0.723	-0.206	-1.003	2.750	-3.273				
		rHipp$_R$	0.498	-0.548	-0.770	4.889	0.936	0.436				
DTI+NODDI+MAP	0.893	cHipp$_L$	-0.677	-0.693	-0.371	-1.269	-1.342	0.861	0.267	-0.833	-0.833	0.019
		cHipp$_R$	0.301	-0.043	0.132	-2.076	-0.753	-1.182	-1.691	-0.430	1.513	-0.068
		rHipp$_L$	-1.982	-0.525	-1.111	-0.392	2.437	-2.235	-0.270	0.325	-0.216	0.295
		rHipp$_R$	-0.430	-0.609	-0.415	3.531	0.529	0.825	0.114	0.511	-0.593	-0.897
NODDI	0.857	cHipp$_L$				-3.065	-2.537	5.261				
		cHipp$_R$				4.383	-0.905	-14.232				
		rHipp$_L$				-1.172	2.443	-8.511				
		rHipp$_R$				10.051	1.926	-0.829				
NODDI+MAP	0.857	cHipp$_L$				-0.323	-1.665	1.462	-0.898	-0.233	-0.579	0.196
		cHipp$_R$				-0.310	-2.251	-2.451	-3.342	-0.406	2.032	-1.301
		rHipp$_L$				-0.686	2.274	-4.742	0.363	1.145	-1.351	0.327
		rHipp$_R$				5.716	0.656	-0.221	-1.494	0.639	-0.294	-0.358
DTI	0.804	cHipp$_L$	0.977	-0.391	-1.058							
		cHipp$_R$	2.130	-0.127	-2.534							
		rHipp$_L$	-5.314	0.958	4.288							
		rHipp$_R$	-2.206	-0.472	1.835							
DTI+MAP	0.732	cHipp$_L$	0.012	-0.029	0.448				-1.065	-0.707	-5.024	1.380
		cHipp$_R$	3.193	0.214	-1.923				-2.879	-0.262	-0.334	1.098
		rHipp$_L$	-7.788	-0.591	5.142				-6.616	0.534	-0.280	1.166
		rHipp$_R$	-1.769	-1.903	-0.632				-1.601	0.616	-4.487	-1.392

Table S1: Coefficients for Hippocampal Diffusion Metrics Predicting Binarized Age in the UCR Dataset

			DTI			NODDI			MAP				
Predictor	AUC	Region	AD	GFA	RD	ODI	NDI	FWF	MSD	QIV	RTOP	NG	volume
NODDI+MAP	0.924000	cHipp$_L$				2.875	-2.081	-3.690	-1.450	0.125	-0.080	-3.777	
		cHipp$_R$				1.748	3.225	-5.035	-2.170	0.119	0.014	-5.554	
		rHipp$_L$				-3.689	0.974	1.983	0.880	0.471	0.530	3.317	
		rHipp$_R$				2.723	3.473	3.213	0.552	2.281	2.430	3.306	
DTI+NODDI	0.884000	cHipp$_L$	5.675	-4.417	-5.096	1.498	-1.923	-1.467					
		cHipp$_R$	3.757	-6.404	-4.014	-1.275	1.653	-3.782					
		rHipp$_L$	-0.896	-1.005	0.876	-3.590	1.729	-0.595					
		rHipp$_R$	10.358	-5.702	-8.989	1.391	4.011	-4.034					
NODDI	0.819000	cHipp$_L$				0.950	-0.491	-1.417					
		cHipp$_R$				-0.191	0.410	-0.643					
		rHipp$_L$				-0.609	0.479	-0.248					
		rHipp$_R$				0.224	0.246	-0.067					
DTI+NODDI+MAP	0.805000	cHipp$_L$	0.085	0.126	-0.196	1.643	-1.821	-1.573	-1.082	0.470	0.405	-2.482	
		cHipp$_R$	-0.345	-1.257	0.651	-0.506	1.919	-2.878	-1.922	0.508	0.478	-3.592	
		rHipp$_L$	-0.266	-1.106	-0.749	-3.064	1.550	1.447	0.535	-0.111	-0.077	2.996	
		rHipp$_R$	2.344	-1.284	-0.812	1.459	3.572	0.647	-0.783	1.103	1.167	1.799	
MAP	0.776000	cHipp$_L$							-1.700	3.122	-3.957	-0.107	
		cHipp$_R$							-0.798	0.888	-0.338	-1.082	
		rHipp$_L$							-0.656	-2.415	1.626	0.981	
		rHipp$_R$							1.433	-1.519	2.269	0.767	
DTI+MAP	0.765000	cHipp$_L$	0.216	-1.455	-0.215				-6.147	-0.305	-2.817	-3.086	
		cHipp$_R$	-0.458	-1.451	3.504				-14.775	-2.616	-3.363	-0.824	
		rHipp$_L$	-0.952	2.761	-1.446				1.520	-1.014	0.353	2.653	
		rHipp$_R$	2.622	-2.183	-2.074				3.328	-0.232	0.480	4.256	

Table S2: Coefficients for Hippocampal Diffusion Metrics Predicting Binarized Age in the ADNI Dataset

Predictor	AUC	Region	DTI			NODDI			MAP				
			AD	GFA	RD	ODI	NDI	FWF	MSD	QIV	RTOP	NG	volume
volume	0.719000	cHipp$_L$											-0.396
		cHipp$_R$											-0.243
		rHipp$_L$											0.340
		rHipp$_R$											-0.019
DTI	0.708000	cHipp$_L$	-0.705	0.072	0.359								
		cHipp$_R$	0.577	-0.947	-1.329								
		rHipp$_L$	-1.521	1.726	1.794								
		rHipp$_R$	1.373	-0.756	-1.679								

Table S2 Cont'd: Coefficients for Hippocampal Diffusion Metrics Predicting Binarized Age in the ADNI Dataset

Predictor	Dataset	F	P	R^2adj
DTI+NODDI	UCR	4.416796	0.000073	0.598549
DTI+NODDI+MAP	ADNI	3.739803	0.000036	0.581107
DTI+MAP	ADNI	4.892359	0.000000	0.579756
NODDI+MAP	ADNI	4.613287	0.000001	0.561530
DTI+NODDI	ADNI	5.190530	0.000000	0.560067
MAP	ADNI	7.007929	0.000000	0.548898
NODDI	ADNI	7.447629	0.000000	0.494793
DTI	UCR	5.272770	0.000019	0.473554
NODDI	UCR	5.132564	0.000018	0.448417
DTI+MAP	UCR	2.565705	0.006989	0.434747
DTI+NODDI+MAP	UCR	1.753947	0.119928	0.354141
MAP	UCR	2.878601	0.002479	0.323000
NODDI+MAP	UCR	1.952954	0.033120	0.304310
DTI	ADNI	3.354187	0.000740	0.263405
volume	UCR	3.118883	0.021444	0.118580
volume	ADNI	0.851446	0.497189	-0.007579

Table S3: Hippocampal Diffusion Predicting Linear Age in the UCR and ADNI Datasets

Predictor	AUC	Region	DTI			NODDI			MAP			
			AD	GFA	RD	ODI	NDI	FWF	MSD	QIV	RTOP	NG
DTI+NODDI+MAP	0.969000	cHipp$_L$	0.299	-0.124	-0.068	0.218	1.917	1.093	0.268	-0.022	-0.088	-0.270
		cHipp$_R$	1.434	3.451	0.392	-0.912	2.753	0.937	-0.138	-0.256	0.475	-0.270
		rHipp$_L$	0.277	0.838	-0.322	-0.876	-2.167	0.692	0.339	-0.253	0.088	0.835
		rHipp$_R$	-0.201	-0.777	-0.328	0.221	-0.510	0.437	0.298	0.986	0.306	-0.308
DTI+NODDI	0.844000	cHipp$_L$	0.155	0.210	-0.438	1.585	1.386	2.874				
		cHipp$_R$	1.489	4.279	0.705	-1.733	3.630	0.170				
		rHipp$_L$	0.441	1.876	-1.273	-0.877	-3.576	1.696				
		rHipp$_R$	-0.594	-1.464	-0.713	0.089	-0.805	0.442				
NODDI	0.775000	cHipp$_L$				1.529	1.998	1.304				
		cHipp$_R$				-11.043	5.546	5.830				
		rHipp$_L$				-4.519	-2.891	4.645				
		rHipp$_R$				1.951	-0.237	0.613				

Table S4: Coefficients for Hippocampal Diffusion metrics predicting RAVLT in the UCR dataset

			DTI			NODDI			MAP			
Predictor	AUC	Region	AD	GFA	RD	ODI	NDI	FWF	MSD	QIV	RTOP	NG
DTI+NODDI	0.790000	cHipp_L	2.048	-3.546	-1.532	-2.724	-0.567	0.168				
		cHipp_R	-2.078	2.539	1.226	1.563	-0.710	0.351				
		rHipp_L	-4.974	3.973	4.914	0.425	-0.289	0.141				
		rHipp_R	-1.158	-0.672	2.639	-1.003	0.859	-1.113				

Table S5: Coefficients for Hippocampal Diffusion Metrics Predicting RAVLT Performance in the ADNI Dataset

			DTI			NODDI			MAP			
Predictor	AUC	Region	AD	GFA	RD	ODI	NDI	FWF	MSD	QIV	RTOP	NG
NODDI	0.751000	cHipp_L				-0.309	0.332	-0.166				
		cHipp_R				-0.873	-1.115	1.666				
		rHipp_L				0.070	0.433	0.222				
		rHipp_R				-0.359	-0.231	-0.404				
NODDI+MAP	0.744000	cHipp_L				-1.750	-0.606	4.601	-20.655	-6.892	-9.541	0.696
		cHipp_R				-1.411	-2.229	-0.571	2.821	-0.323	0.650	-1.082
		rHipp_L				0.070	0.755	0.795	1.216	1.302	-0.165	0.811
		rHipp_R				-0.342	-0.252	-2.134	5.220	3.352	-0.865	2.134

Table S6: Coefficients for Hippocampal Diffusion Metrics Predicting Trails B Performance in the ADNI Dataset

			DTI			NODDI			MAP				
Predictor	AUC	Region	AD	GFA	RD	ODI	NDI	FWF	MSD	QIV	RTOP	NG	volume
DTI	0.740000	cHipp_L	2.647	-1.416	-1.687								
		cHipp_R	-3.502	3.139	3.795								
		rHipp_L	1.264	-0.985	-0.997								
		rHipp_R	0.614	-0.688	-1.234								
DTI+NODDI	0.740000	cHipp_L	4.298	-1.862	-3.219	-2.078	-2.317	2.692					
		cHipp_R	-3.879	1.719	5.021	-0.108	2.780	-3.240					
		rHipp_L	3.274	-4.096	-2.142	-1.058	0.690	-0.814					
		rHipp_R	1.755	-0.045	-4.115	1.567	-0.834	2.055					
NODDI	0.702000	cHipp_L				-0.593	-0.820	0.747					
		cHipp_R				0.516	0.978	-1.072					
		rHipp_L				0.249	0.024	-0.134					
		rHipp_R				0.235	-0.174	0.191					
volume	0.694000	cHipp_L											-0.585
		cHipp_R											0.260
		rHipp_L											-0.230
		rHipp_R											0.612

Table S7: Coefficients for Hippocampal Diffusion Metrics Predicting CBB One-Back Performance in the ADNI Dataset

			DTI			NODDI			MAP				
Predictor	AUC	Region	AD	GFA	RD	ODI	NDI	FWF	MSD	QIV	RTOP	NG	volume
DTI+MAP	0.672	NAC_R	0.806	-0.106	-0.508				-0.483	-11.336	11.011	0.142	
NODDI+MAP	0.646	NAC_R				-0.407	-0.439	0.814	-1.501	-11.526	10.514	0.113	
DTI	0.583	NAC_R	0.638	-0.095	-0.575								
DTI+NODDI	0.500	NAC_R	0.497	0.011	-0.294	-0.026	0.003	-0.199					
DTI+NODDI+MAP	0.500	NAC_R	0.783	-0.399	-0.469	-0.448	-0.334	0.741	-1.930	-12.003	10.640	0.235	
MAP	0.500	NAC_R							-0.140	-7.982	7.948	-0.066	
NODDI	0.500	NAC_R				-0.275	-0.091	0.029					
volume	0.500	NAC_R											0.023

Table S8: Coefficients for Right NAC Diffusion Metrics Predicting Trails B Performance in the ADNI Dataset

			DTI			NODDI			MAP				
Predictor	AUC	Region	AD	GFA	RD	ODI	NDI	FWF	MSD	QIV	RTOP	NG	volume
DTI+MAP	0.808	NAC_R	1.406	-1.462	-1.685				-1.433	1.692	-4.360	0.718	
DTI+NODDI+MAP	0.779	NAC_R	1.526	-1.467	-1.686	0.143	0.372	-0.685	-0.363	1.825	-3.865	0.752	
MAP	0.769	NAC_R							-0.835	-2.190	0.445	0.579	
NODDI+MAP	0.760	NAC_R				0.561	0.318	-1.052	0.364	1.136	-2.421	0.579	
NODDI	0.740	NAC_R				0.492	0.260	-0.037					
DTI+NODDI	0.740	NAC_R	-0.114	0.048	0.309	0.453	0.381	-0.188					
volume	0.713	NAC_R											-0.060
DTI	0.692	NAC_R	-0.693	0.145	0.693								

Table S9: Coefficients for Right NAC Diffusion Metrics CBB One-Back Performance in the ADNI Dataset
